# Dementia in Southeast Asia: influence of onset-type, education, and cerebrovascular disease

**DOI:** 10.1186/s13195-021-00936-y

**Published:** 2021-11-30

**Authors:** Ashwati Vipin, Vaynii Satish, Seyed Ehsan Saffari, Wilbur Koh, Levinia Lim, Eveline Silva, Mei Mei Nyu, Tanya-Marie Choong, Esther Chua, Linda Lim, Adeline Su Lyn Ng, Hui Jin Chiew, Kok Pin Ng, Nagaendran Kandiah

**Affiliations:** 1 National Neuroscience Institute, 11 Jalan Tan Tock Seng, Singapore, 308433 Singapore; 2 Duke-NUS Medical School, Singapore, Singapore; 3 Lee Kong Chian School of Medicine, Nanyang Technological University, Singapore, Singapore

**Keywords:** Longitudinal, Clinical, Alzheimer’s disease, Cognition, Education, Cerebrovascular disease, Southeast Asian cohort

## Abstract

**Background:**

Southeast Asia represents 10% of the global population, yet little is known about regional clinical characteristics of dementia and risk factors for dementia progression. This study aims to describe the clinico-demographic profiles of dementia in Southeast Asia and investigate the association of onset-type, education, and cerebrovascular disease (CVD) on dementia progression in a real-world clinic setting.

**Methods:**

In this longitudinal study, participants were consecutive series of 1606 patients with dementia from 2010 to 2019 from a tertiary memory clinic from Singapore. The frequency of dementia subtypes stratified into young-onset (YOD; <65 years age-at-onset) and late-onset dementia (LOD; ≥65 years age-at-onset) was studied. Association of onset-type (YOD or LOD), years of lifespan education, and CVD on the trajectory of cognition was evaluated using linear mixed models. The time to significant cognitive decline was investigated using Kaplan-Meier analysis.

**Results:**

Dementia of the Alzheimer’s type (DAT) was the most common diagnosis (59.8%), followed by vascular dementia (14.9%) and frontotemporal dementia (11.1%). YOD patients accounted for 28.5% of all dementia patients. Patients with higher lifespan education had a steeper decline in global cognition (*p*<0.001), with this finding being more pronounced in YOD (*p*=0.0006). Older patients with a moderate-to-severe burden of CVD demonstrated a trend for a faster decline in global cognition compared to those with a mild burden.

**Conclusions:**

There is a high frequency of YOD with DAT being most common in our Southeast Asian memory clinic cohort. YOD patients with higher lifespan education and LOD patients with moderate-to-severe CVD experience a steep decline in cognition.

**Supplementary Information:**

The online version contains supplementary material available at 10.1186/s13195-021-00936-y.

## Background

The prevalence of dementia types and their clinical trajectory has been extensively reported from western settings [1–9]. The estimates from Asia are quite variable, likely related to differing diagnostic criteria and variations in diagnostic tools. There is also limited literature on dementia progression in Asia, especially involving naturalistic patient follow-up over long durations. Longitudinal studies in Asia have largely focussed on older populations [10] and cognitive changes in non-dementia participants [11]. Cognitive trajectories in young-onset dementia (YOD) have not been compared with late-onset dementia (LOD) counterparts in Asia [12–14]. Additionally, the prevalence of dementia sub-types remains to be elucidated in Southeast Asia.

From an etiological perspective, reports illustrate cerebrovascular contribution to dementia in Asia [15, 16], involving white matter hyperintensities (WMH) as surrogate MRI markers for cerebrovascular disease (CVD) [17]. While vascular cognitive impairment (VCI) is more prevalent in Asia [18, 19], there is limited data from naturalistic studies comparing cognitive trajectories in such patients. Additionally, the contribution of education attainment to cognitive decline between YOD and LOD remains to be further elucidated.

This study describes the demographic and clinical trends and longitudinal profile of YOD and LOD patients from a dementia clinic of a tertiary hospital, in Singapore between 2010 and 2019. The tertiary hospital is the largest provider of neuroscience care in Singapore, providing care to 70% of the population. Additionally, the study examined the association between education and longitudinal cognitive change in YOD and LOD. The impact of CVD determined by the modified Fazekas scale on clinically relevant time to a significant decline in global cognition was examined, as this may allow clinicians to institute intensive management of vascular risk factors for those with CVD. We also assessed the influence of education and CVD on depression. We hypothesized that higher levels of lifespan education would be protective against cognitive decline and higher CVD load would result in a more rapid cognitive decline.

## Methods

### Participants and study design

Data was extracted from a longitudinal database of consecutive series of patients with cognitive impairment. The database included patients attending the dementia clinic of a tertiary hospital in Singapore between 2010 and 2019. All patients were evaluated by a team comprising cognitive neurologists, psychologists, and dementia-trained nurses. Patients underwent neuroimaging with MRI or a CT scan as part of the diagnostic workup where clinically indicated. Diagnoses included subjective cognitive impairment, mild cognitive impairment, and dementia. For the purposes of this study, only patients with dementia were included in the analysis. Dementia was diagnosed based on the DSM IV and 5 criteria [20, 21]. Patients with dementia included dementia of the Alzheimer’s type (DAT), frontotemporal dementia (FTD), vascular dementia (VaD), Parkinsonism spectrum dementia, rapidly progressing dementia (RPD), and autoimmune dementia. Clinical symptoms and presentation largely informed the diagnoses in the case of mixed dementias, wherein the consulting team arrived at a final diagnosis based on the presentation and clinical history of individual patients. Parkinsonism spectrum dementia consisted of Parkinson’s dementia (PDD), dementia with Lewy body (DLB), and normal pressure hydrocephalus (NPH); diagnosis of DAT was per the NINCDS-ADRDA [22] and NIA-AA criteria [23]. VaD was diagnosed based on the NINDS-AIREN criteria [24], FTD was diagnosed based on the Raskovsky criteria [25], PDD was diagnosed based on the MDS task force criteria [26, 27], while DLB was diagnosed based on the McKeith criteria [28, 29]. Patients who were under the age of 65 years at the time of symptom onset were classified as YOD [30], while patients aged 65 years or older were classified as LOD. A total of 2890 unique patients attended the tertiary memory clinic from 2010 to 2019. Of them, 1606 were given a consensus diagnosis of dementia based on clinical criteria (as referenced above) on their initial visit, a subset of whom came back for follow-up visits every 6 to 9 months. At every follow-up visit, patient diagnosis and demographics were determined and they underwent cognitive testing as detailed in section 2.2. Dementia patients were included in the study if they had at least one follow-up visit following the initial visit and based on these criteria a total of 786 dementia patients completed one or more follow-up visits. As part of the routine clinical screening, patients were evaluated for thyroid function and vitamin B12 levels at every visit. While we have not collected individual data on these measures, appropriate management was employed for patients with any abnormal findings.

The study was approved by the Singhealth Centralized Review Board. The informed consent process was in accordance with Declaration of Helsinki and local clinical research regulations.

### Cognitive and behavioral measurements

Patients underwent evaluation for global cognition with the local versions of the Mini Mental State Examination (MMSE) and the Montreal Cognitive Assessment (MoCA), which have been validated in Singapore [31, 32]. The local version of the MMSE and MOCA has a score range of 0–30 points and score categories similar to the original versions. Patients were also screened for depression with the geriatric depression scale (GDS). Clinically relevant depression was determined using a cut-off of GDS≥5. The MMSE and MoCA evaluation was conducted at the patient’s initial visit to the clinic and was classified as the baseline MMSE and baseline MoCA score. These evaluations were then repeated at every clinical visit which ranged from 6 to 9 months and were used as follow-up measurements in the statistical analyses. The MMSE was the most regularly recorded measure due to the ease of administration with limited patient-facing time in a naturalistic clinical setting. It was also an effective tool for consistent tracking of cognitive performance as disease progression did not hinder the administration of the test until more advanced stages. All evaluations were performed by psychologists and trained dementia nurses using a standardized protocol.

### Measurement of cerebrovascular disease burden

Patients underwent neuroimaging using either 1.5T MRI scanner (Philips Ingenia) or 3T Siemens Prisma Fit (Siemens, Erlangen, Germany) or coronal fine cut CT scans. T1-weighted and fluid-attenuated inversion recovery were used for visual rating of scans based on the modified Fazekas scale for WMH severity [33]. Specifically, periventricular WMH and deep subcortical WMH were separately rated on a 0–3 point scale for both hemispheres. The scoring criteria were as follows: for periventricular WMH, the absence of any WMH = 0; the presence of caps or pencil-thin lining = 1; a smooth halo along the edges of the lateral ventricle = 2; and irregular hyperintensities extending into deep white matter = 3. For deep subcortical WMH, the absence of any WMH = 0; the presence of non-confluent foci of WMH in the deep subcortical region = 1; beginning confluence of WMH foci = 2; and the presence of large confluent areas = 3. The modified Fazekas scale allows for quantification of white matter lesions in four brain regions, namely right periventricular, left periventricular, right deep subcortical, and left deep subcortical to provide a score range of 0–12. All visual ratings were performed by two independent raters, and any significant discordance in scores was resolved by consensus. Absent-to-mild CVD was defined as a score of 0–4 on the total Fazekas scale while those with a score of 5–12 were defined as moderate-to-severe CVD. A score of 5–12 on the modified Fazekas score corresponds to Fazekas grades 2–3 of the original grading, indicative of significant CVD.

### Statistical analyses

Analyses of baseline demographic information for the dementia group included descriptive statistics, and continuous variables were reported as mean and standard deviation and categorical variables as frequency and percentage across YOD and LOD cohorts. Group differences were examined using two independent samples *t* test or Mann-Whitney *U* test (where appropriate) and *χ*^2^ or Fisher’s exact test (where appropriate) for continuous and categorical outcomes, respectively.

In order to investigate the cognitive trajectories of YOD and LOD over time (duration of follow-up), linear mixed model analysis was conducted to examine two-way interactions between onset-type and time, for patients with at least one follow-up MMSE score. Longitudinal MMSE scores were the key dependent variable. The key independent variable was onset-type, coded as either YOD (<65 years) or LOD (≥65 years) and an onset-type*Time interaction term. Baseline age, race, sex, and lifespan education years (referring to education from primary school and throughout the lifespan) and duration of follow-up were included as covariates. Additionally, a separate model also assessed the effect of lifespan education years on MMSE decline over time with a lifespan education years*time interaction term as the key independent variable. Baseline age, race, sex, baseline MMSE score, and duration of follow-up were included as covariates. The random effects were modeled at the individual subject level represented by the individual variability in intercepts and longitudinal slopes.

Additionally, the relationship between onset-type, lifespan education, and cognition was elucidated in a linear mixed model three-way interaction analysis. The key dependent and independent variables remained longitudinal MMSE scores and onset-type respectively with an onset-type*lifespan education years*time interaction term. Baseline age, sex, race, lifespan education years, baseline MMSE score, and duration of follow-up were included as covariates. The random effects were modeled at the individual subject level represented by the individual variability in intercepts and longitudinal slopes.

The linear mixed model analyses were performed using R 3.6.3 (R Core Team, 2014) with RStudio (RStudio Team, 2012).

A subset of our patients also had longitudinal GDS scores. Thus, we also investigated what factors contributed to the onset of clinically relevant depression or the continued presence of depression using a cut-off of GDS≥5. Based on this cut-off, we classified patients on a binary scale (0=not depressed; 1=depressed). In binary logistic regression models, we assessed whether lifespan education years, sex, and white matter hyperintensity (Fazekas visual rating scores) influenced the development of depression in YOD and LOD separately.

In order to examine a clinically relevant effect of CVD burden on the rapid progression of cognitive decline, a sub-group analysis looking at the long-term effect of CVD burden on those diagnosed with DAT and VaD was conducted. We investigated the effect of CVD burden on the significant decline in MMSE and MoCA using a Kaplan-Meier plot and log-rank test. For MMSE and MoCA scores, a three-point drop was used to define significant decline within the maximum duration of follow-up for each patient. As defined earlier, patients with absent-to-mild cerebrovascular disease (CVD) had a score of 0–4 on the total Fazekas scale while those with a score of 5–12 were defined as moderate-to-severe CVD. The sub-group statistical analysis was conducted using SAS software version 9.4 for Windows (Cary, NC: SAS Institute Inc.) and R 3.6.3 (R Core Team, 2014) with RStudio (RStudio Team, 2012).

## Results

### Demographics of dementia

A total of 2890 unique patients attended the tertiary memory clinic from 2010 to 2019. Of them, 1606 were diagnosed with dementia. The mean age of the dementia cohort was 71.2 (± 10.5) years, 53.9% females, and 85.4% Chinese ethnicity with a mean lifespan education of 7.4 (± 5.2) years. DAT was the most common diagnosis (59.8%), followed by VaD (14.9%), FTD (11.1%), Parkinsonism spectrum (11.1%), autoimmune dementia (1.6%), and RPD (1.4%). YOD patients accounted for 28.5% of all dementia patients. Over the 10-year period, there was an increasing trend in the yearly incidence of YOD and LOD patients (Additional File 1: Supplementary Fig. 1).

### Clinical profiles of young-onset and late-onset dementia

**Fig. 1:**
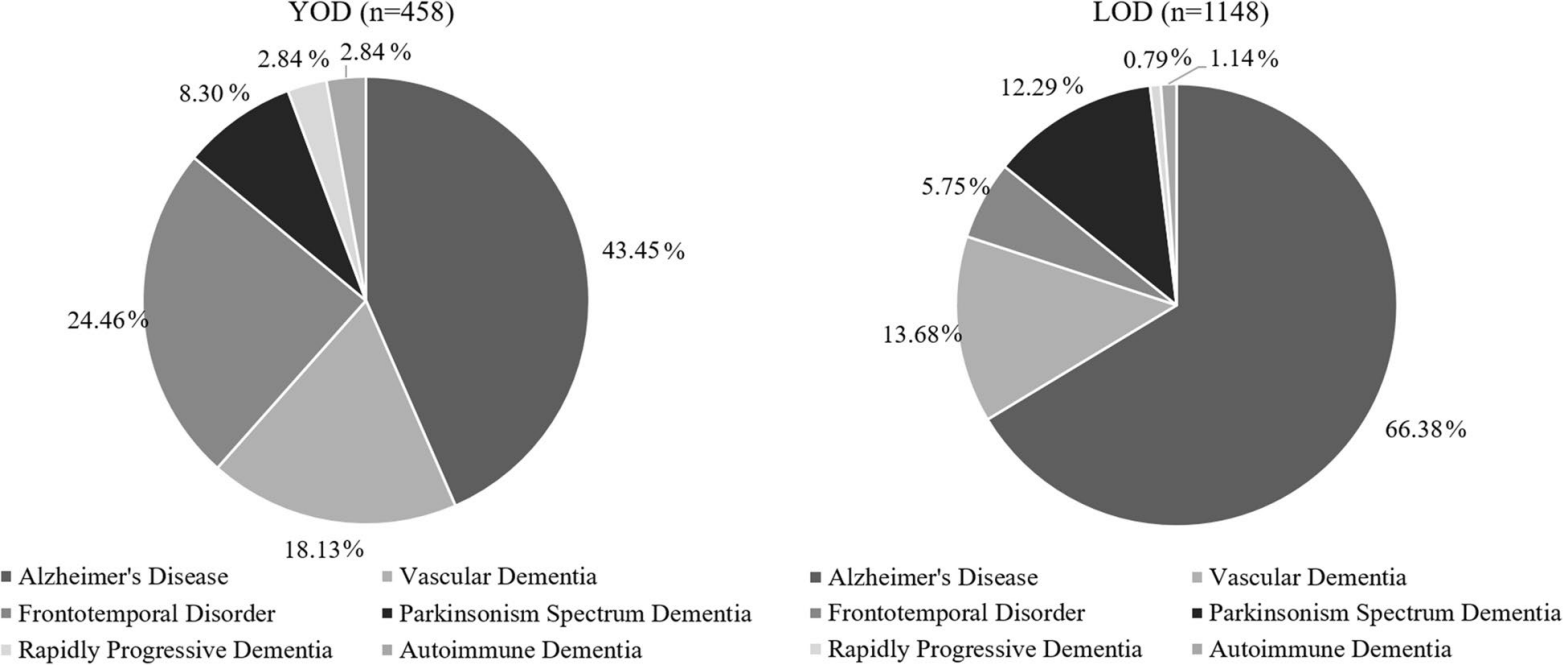
Diagnostic breakdown in young-onset dementia and late-onset dementia. Dementia of the Alzheimer’s type was the most common diagnosis in both the young-onset and late-onset groups. The late-onset dementia group had a significantly higher proportion of dementia of the Alzheimer’s type patients. Abbreviations: YOD young-onset dementia, LOD late-onset dementia

Patients in the LOD group had significantly lower years of lifespan education (6.5 vs. 9.6, *p*<0.001), and lower baseline MMSE (17.7 vs. 18.9, *p*=0.003) and MoCA (16.8 vs. 18.1, *p*=0.002) compared to YOD patients (Table 1). LOD participants also had a higher frequency of diabetes mellitus (22.2 vs. 15.7, *p*=0.002), hypertension (46.8 vs. 29.7 *p*<0.001), hyperlipidemia (42.1 vs. 31.2, *p*<0.001), and coronary artery disease (8.8 vs. 3.9, *p*=0.001) but lower frequency of smoking (12.6 vs 15.5, *p*=0.010) and alcohol use (7.9 vs. 12.0, *p*=0.007) (Table 1). The LOD group had a significantly higher proportion of DAT patients (66.4% vs. 43.5%, *p*<0.001) and Parkinsonism spectrum patients (12.3% vs. 8.3%, *p*=0.028) compared to the YOD group (Table 1). However, there was a significantly higher proportion of VaD patients (18.1% vs. 13.7%, *p*=0.029), FTD patients (24.5% vs. 5.8%, *p*<0.001), RPD patients (2.8% vs. 0.8%, *p*=0.003), and autoimmune dementia patients (2.8% vs. 1.1%, *p*=0.026) in the YOD than the LOD group (Table 1; Fig. 1).

**Table 1:** Baseline demographics and clinical characteristics by young and late-onset dementia

Variable	Young-onset dementia (***n***=458)	Late-onset dementia (***n***=1148)	***P value***^**a**^
**Demographics**			
Sex (female), *n* (%)	215 (47.0)^b^	651 (56.7)	<0.001
Race (Chinese), *n* (%)	359 (78.4)	1012 (88.2)	<0.001
Age (year ± SD)	58.0 ± 5.9	76.48 ± 6.6	<0.001
Years of education (year ± SD)	9.6 ± 4.7	6.52 ± 5.2	<0.001
**Baseline clinical history**			
Diabetes, *n* (%)	72 (15.7)	255 (22.2)	0.002
Hypertension, *n* (%)	136 (29.7)	537 (46.8)	<0.001
Hyperlipidemia, *n* (%)	143 (31.2)	483 (42.1)	<0.001
Smoking, *n* (%)	71 (15.5)	145 (12.6)	0.010
Alcohol intake, *n* (%)	55 (12.0)	91 (7.9)	0.007
Coronary artery disease, *n* (%)	18 (3.9)	101 (8.8)	0.001
Atrial fibrillation, *n* (%)	4 (0.9)	27 (2.4)	0.074
Family history of dementia, *n* (%)	68 (14.9)	106 (9.2)	0.002
**Cognitive measures**			
Mini Mental State Examination (MMSE score ± SD)	18.9 ± 6.9	17.7 ± 6.1	0.003
Montreal Cognitive Assessment (MoCA score ± SD)	18.1 ± 6.2	16.8 ± 5.1	0.002
MMSE-annual decline, score ± SD	2.1 ± 6.9	1.5 ± 6.1	0.233
MoCA-annual decline, score ± SD	1.2 ± 6.2	0.6 ± 5.1	0.315
Geriatric Depression Scale (GDS) (≥5)	80 (17.5)	209 (18.2)	0.726
**Dementia sub-types**			
Alzheimer’s type dementia, *n* (%)	199 (43.5)	762 (66.4)	<0.001
Vascular dementia, *n* (%)	83 (18.1)	157 (13.7)	0.029
Frontotemporal dementia, *n* (%)	112 (24.5)	66 (5.8)	<0.001
Parkinsonism spectrum^1^, *n* (%)	38 (8.3)	141 (12.3)	0.028
Rapidly progressing dementia, *n* (%)	13 (2.8)	9 (0.8)	0.003
Autoimmune dementia, *n* (%)	13 (2.8)	13 (1.1)	0.026

^a^Chi-square or Fisher’s exact test (where appropriate) for categorical variables; two independent samples *t* test or Mann-Whitney *U* test (depending on normality assumption) for continuous variables

^b^Continuous variables reported as mean ± standard deviation; categorical variables reported as frequency (percentage)

^1^Parkinsonism spectrum is inclusive of PD, DLB, and NPH

### Effect of onset-type and education on cognitive trajectories of patients with amnestic-type dementia

Change in global cognition indexed using MMSE was studied in 786 patients with DAT and VaD having follow-up MMSE scores. There were 194 patients (24.7%) in the YOD group with a mean age of 58.4 (± 5.0) years, mean lifespan education of 9.3 (± 4.0) years, 47.9% of females, 80.9% of Chinese ethnicity, baseline MMSE of 19.8 (± 5.8), and baseline MoCA of 17.8 (± 5.7). There were 592 (75.3%) LOD patients with a mean age of 76.5 (± 5.9), an average of 6.2 (± 5.0) years of lifespan education, 62.7% female and 90.4% Chinese ethnicity, and baseline MMSE of 18.6 (± 5.5) and baseline MoCA of 16.7 (± 4.9). The average duration of follow-up for the YOD group was 2.3 (± 1.8) years and 2.8 (± 2.0) years for the LOD group.

YOD patients, while demonstrating a higher MMSE score at baseline compared to the LOD group (19.8 vs. 18.6, *p*=0.014), showed a steeper decline in MMSE scores over a mean follow-up time of 2.7 years (onset-type*Time: *β*=−0.33, *p*=0.030; Additional File 1: Supplementary Fig. 2; Table 2) compared to LOD patients after controlling for age, sex, race, and lifespan education years.

**Table 2:** Summary of linear mixed-effects models examining the independent and interactive effects of onset-type, lifespan education, and time on longitudinal MMSE decline and summary of binary logistic regression models examining the effect of lifespan education, sex, and white matter hyperintensity on depression development in YOD and LOD

	***β*** **estimate**	***t***	***P value***
Effect of onset-type on longitudinal MMSE decline (linear mixed-effects model) MMSE ~ baseline age + sex + race + onset-type + time + onset-type*time			
Onset-type	−1.57	−2.301	0.021
Time	−1.01	−14.573	<0.0001
Onset-type*Time	−0.33	−2.218	0.030
**Effect of lifespan education on longitudinal MMSE decline (linear mixed-effects model)** MMSE ~ baseline age + sex + race + lifespan education + time + lifespan education*time			
Lifespan education	0.39	9.988	<0.0001
Time	−0.73	−7.032	<0.0001
Lifespan education*Time	−0.05	−4.178	<0.0001
Interaction effect of onset-type and lifespan education on longitudinal MMSE declineMMSE ~ baseline age + sex + race + onset-type + lifespan education + time + onset-type*Time + lifespan education*time + onset-type*lifespan education*time			
Onset-type	−1.86	−1.696	0.09
Lifespan education	0.38	9.054	<0.0001
Time	−0.81	−7.629	<0.0001
Onset-type*lifespan education	0.03	0.314	0.7538
Onset-type*time	0.95	2.600	0.0096
Lifespan education*time	−0.03	−2.31	0.0214
Onset-type*lifespan education*time	−0.13	−3.414	0.0006
**Predictors of depression in YOD (binary logistic regression)**			
	***β*** **estimate**	***z***	***P value***
Lifespan Education	0.016	0.284	0.7761
Sex	−0.487	−1.034	0.3013
White matter hyperintensity (Fazekas visual rating score)	0.049	0.481	0.6306
**Predictors of depression in LOD (binary logistic regression)**			
Lifespan education	−0.044	−1.646	0.0997
Sex	−0.405	−1.007	0.314
White matter hyperintensity (Fazekas visual rating score)	0.065	1.246	0.213

Time refers to years since baseline visit

Abbreviations: *MMSE* mini-mental state examination, *YOD* young-onset dementia, *LOD* late-onset dementia

Years of lifespan education were found to be a predictor of baseline MMSE (*p*<0.001) such that higher years of education resulted in higher baseline MMSE scores. Despite the higher baseline MMSE scores, higher years of education related in a steeper decline in MMSE scores over time (years of education*Time: *β*=−0.05, *p*<0.001; Table 2) after controlling for age, race, and sex. These results remained even after controlling for baseline MMSE score.

**Fig. 2:**
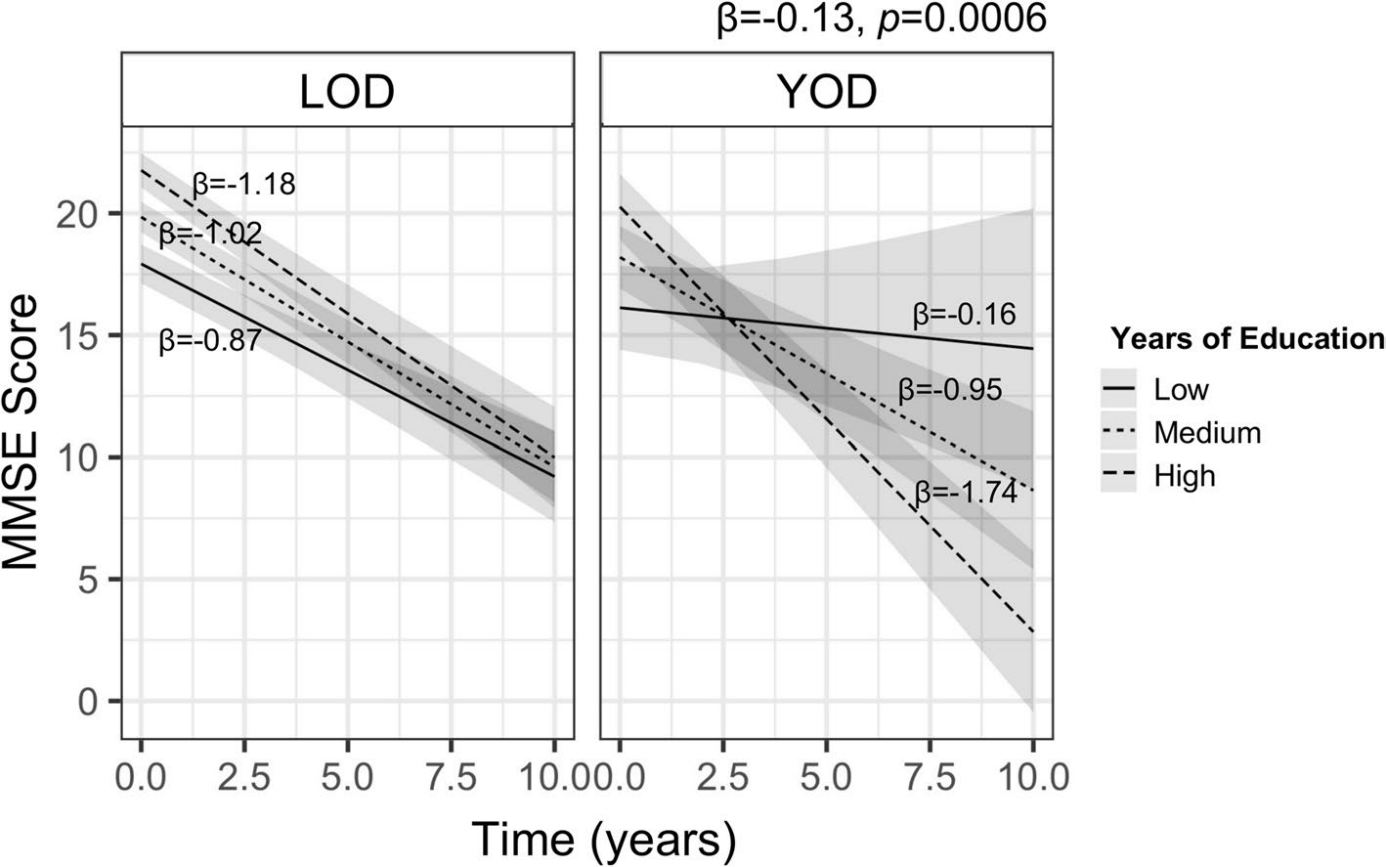
Mini Mental State Examination scores over time for young and late-onset dementia with low, medium, and high education. Patients with higher years of education in the young-onset dementia group experienced a steeper decline than patients with comparable years of education in the late-onset dementia group. Abbreviations: YOD young-onset dementia, LOD late-onset dementia, MMSE Mini Mental State Examination

When onset-type was added in the model evaluating lifespan education and MMSE trajectory, the effect of years of education was found to be more pronounced. Patients with higher years of education in the YOD group experienced a steeper decline than patients with comparable years of education in the LOD group (onset-type*Lifespan education years*Time: *β*=−0.13, *p*=0.0006; Fig. 2, Table 2). At baseline, there was no statistical difference in the MMSE score between those in the YOD and LOD groups with comparable years of education (*p*=0.812). Importantly, these results remained even after controlling for baseline MMSE score.

### Longitudinal depression in young-onset and late-onset dementia

From the total cohort, a subset of 430 patients had longitudinal GDS scores comprising YOD (*n*=104) and LOD (*n*=326) patients. In our memory clinic, we found that 23.07% (*n*=24) of our YOD and 23.33% (*n*=76) of our LOD patients were classified as having depression at follow-up. In separate binary logistic regression models for YOD and LOD, lifespan education years, sex, and white matter hyperintensity (Fazekas visual rating scores) were not significant predictors of depression development (Table 2).

### Influence of cerebrovascular disease burden on a global cognitive trajectory in DAT and VaD

The influence of CVD on global cognitive MMSE trajectory was studied among 592 DAT and VaD YOD and LOD patients who had baseline MRI or CT brain. These patients had a baseline MRI scan for quantification of CVD and longitudinal MMSE scores. CVD burden was coded as a categorical variable, absent-to-mild CVD burden and moderate-to-severe CVD burden as described in the “Methods” section. A three-point drop in MMSE was used to define a significant decline in global cognition [34].

**Fig. 3:**
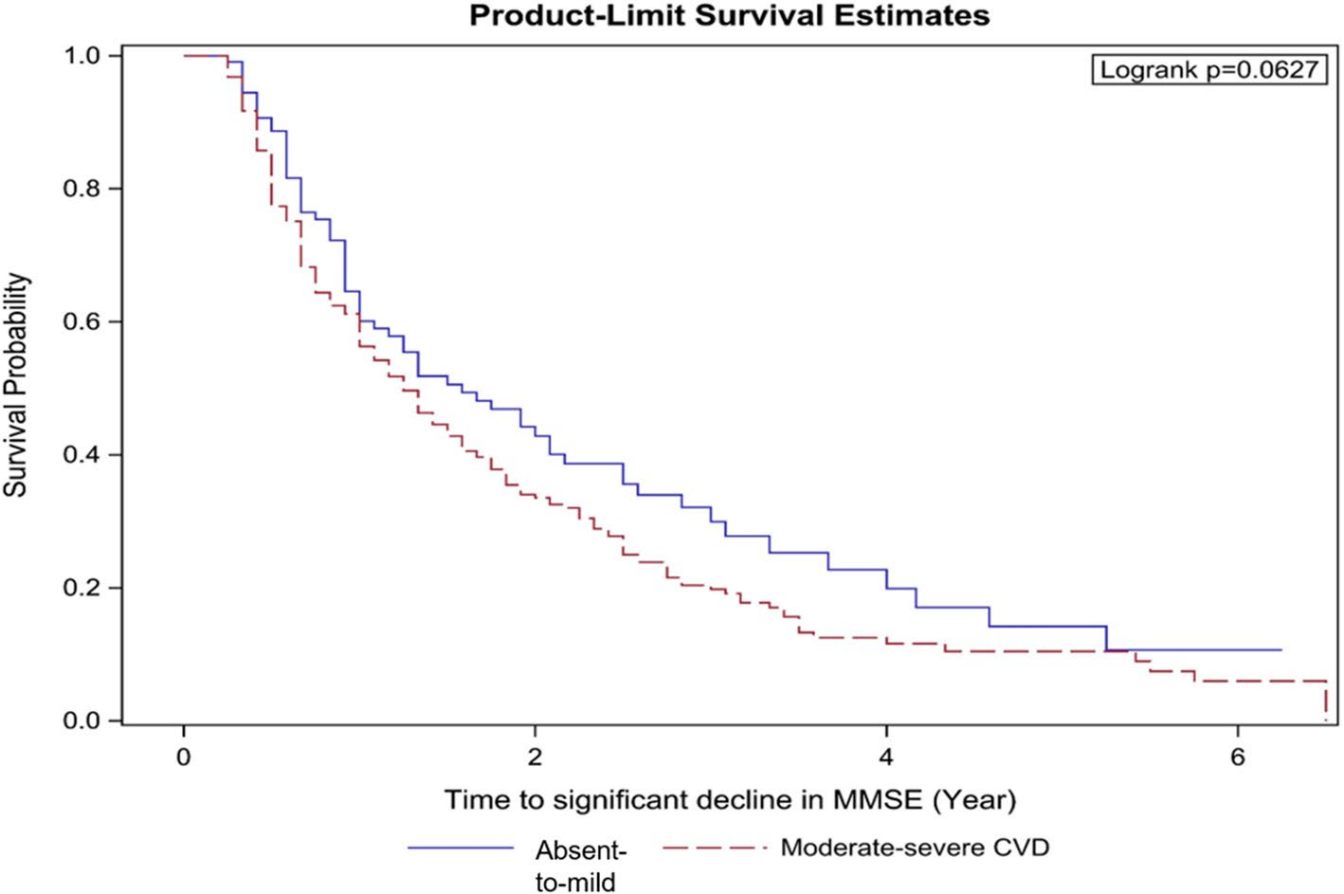
Time to a significant decline in Mini Mental State Examination scores for those with moderate-to-severe cerebrovascular burden and absent-to-mild cerebrovascular burden. Seventy-five percent of late-onset patients with moderate-to-severe cerebrovascular burden, demonstrated a 3-point MMSE decline at 2.5 years, while it took 3.6 years for 75% of patients with an absent-to-mild cerebrovascular burden to have a similar MMSE decline. Abbreviations: YOD young-onset dementia, LOD late-onset dementia, MMSE Mini Mental State Examination

Among the 592 patients, 417 (70.4%) had a moderate-to-severe burden of CVD while 175 (29.6%) had an absent-to-mild burden of CVD. Kaplan-Meier analysis demonstrated that patients in the LOD group with the moderate-to-severe burden of CVD demonstrated a statistical trend for faster decline compared to those with absent-to-mild CVD. Among LOD patients with moderate-to-severe CVD, 75% of them demonstrated a 3-point MMSE decline at 2.5 years, while it took 3.6 years for 75% of LOD patients with absent-to-mild CVD to have a similar MMSE decline (*p*=0.063; Fig. 3). There was no significant difference in time to cognitive decline among YOD patients based on CVD severity (*p*=0.715).

Additionally, a subset of patients also had longitudinal MoCA scores and the influence of CVD on MoCA trajectory was studied among 368 YOD and LOD patients who had baseline MRI or CT brain. There was no significant difference in the average time to 3-point MoCA decline between the absent-to-mild CVD group (2.09 years) and the moderate-to-severe CVD group (2.03 years, *p*=0.69). Additionally, there was also no significant difference in a significant decline in MoCA scores based on CVD severity in both the YOD and LOD groups.

## Discussion

In a Southeast Asian memory clinic cohort with data spanning a decade, we found a trend towards increasing yearly incidence of both YOD and LOD. We additionally demonstrate that YOD accounted for 28.5% of all dementia patients. DAT was the leading type of dementia as in western cohorts, with VaD being the second leading cause of dementia. Our data also showed that while patients with higher education have higher baseline global cognition, they however have a steeper decline in global cognition, with this finding being more pronounced in YOD patients. LOD patients with moderate-to-severe CVD burden also displayed a faster decline in global cognition compared to those with absent-to-mild CVD.

Over the period from 2010 to 2019, overall, there has been an increasing trend in patients with YOD. The reasons for this increase are likely to be multifactorial. Specifically, awareness of YOD both among the general public and within the healthcare system has steadily increased owing to dementia awareness campaigns by many organizations including the Health Promotion Board and the National Neuroscience Institute. The increase in YOD may also be related to the increasing burden of vascular risk factors. Our findings demonstrate that there is a high frequency of vascular risk factors among younger patients. The frequency of hypertension among YOD was 29.7%, while the frequency of hyperlipidemia, diabetes mellitus, and smoking in YOD was 31.2%, 15.7%, and 15.5%, respectively. These vascular risk factors may have contributed to cerebrovascular disease via vascular injury to the brain as well as accelerated amyloid pathology [35–37]. Recent studies in Asian and Western cohorts have also indicated a significant role of vascular disease burden in YOD [38–40]. In support of our findings, prior studies have shown a high prevalence of vascular risk factors and cerebrovascular disease burden in Singapore and other cities in Asia [19, 41]. In turn, high vascular disease burden has been shown to increase the odds of conversion from a prodromal to clinical dementia stage in patients from Singapore [42].

Our findings additionally demonstrate that LOD patients with a moderate-to-severe burden of CVD experience a faster decline in global cognition compared to those with an absent-to-mild CVD burden. Among LOD patients with moderate-to-severe CVD, 75% demonstrated a 3-point decline in MMSE within 2.5 years from diagnosis of dementia, compared to patients with absent-to-mild CVD, wherein a 3-point MMSE decline was observed after 3.6 years These findings suggest that CVD may have a major role in the pathogenesis of dementia in LOD. In this regard, findings from Asia indicate a high co-occurrence of CVD in LOD [43]. Such a co-occurrence and additional presence of vascular risk factors in Asian cohorts have also been associated with greater memory and executive function decline and worse clinical outcomes [44–48]. Additionally, greater dysconnectivity in the default mode network as well as executive control network with consequent impairment in episodic memory as well as executive function has been associated with CVD burden in studies from Asia [46, 49, 50]. Findings from our group demonstrate that the presence of CVD may also result in atrophy of specific gray matter regions crucial for episodic memory and executive function [51, 52]. Moreover, in the Honolulu-Asia Ageing Study, dementia frequency increased with increasing neuritic plaque density and increased further in the presence of cerebrovascular lesions. The association was strongest in patients with sparse neuritic plaques where dementia frequency more than doubled with coexistent cerebrovascular lesions [53]. These findings suggest that CVD may result in accelerated accumulation of amyloid pathology. As for the stronger CVD effect in LOD compared to YOD, it is likely that CVD interacts with the multiple pathologies in the older brain to result in more accelerated neurodegeneration, hence the faster decline in cognition in LOD patients harboring CVD. From a clinical management perspective, our finding of more rapid cognitive decline in patients with concomitant CVD may help clinicians in selecting intensive management of vascular risk factors to slow the rate of decline in their patients with a high CVD burden.

The finding that longitudinal cognitive decline in YOD patients was steeper, especially among those with a higher number of years of lifespan education has important clinical and public health implications. Similar to our findings, previous studies have also reported a steeper decline in cognition among those with higher education [54, 55]. Higher levels of education have been reported to increase cognitive reserve and thus hypothesized to delay the onset of dementia. However, it is likely that once dementia sets in, patients with higher education may have exhausted their cognitive reserve and thus experience a steeper decline in cognition compared to those with lower education. Additionally, YOD is also associated with pure pathologies affecting specific brain regions with a more aggressive disease course, resulting in greater neuronal loss and cerebral hypometabolism than LOD. Thus, the protective role of education is unable to sustain optimal cognitive function in YOD and hence they experience a greater cognitive decline [56–58]. This is an indication that more resources may need to be devoted to the care, treatment, and research for young-onset dementia. Findings from our group indicate that the opportunity cost to the young-onset group in terms of economic, social, and emotional well-being may be more drastic coupled with ripple effects that impact family members of those with young-onset dementia [59, 60]. Notably, little is known about the relationship between onset-type, lifespan education, and cognitive decline in Asian populations and needs further exploration. Findings from our study thus provide important insights into the influence of lifespan education on the future cognitive decline between YOD and LOD in an Asian clinic cohort.

### Limitations

The limitations of our paper include that the data came from a single memory clinic and thus may lack generalizability. However, this being the first report from a memory clinic population, we believe our findings will allow other centers in Southeast Asia to examine their cohorts and perform comparisons with our cohort. The lack of biomarker data may be viewed as a weakness, although the availability of clinical data may allow better allocation of resources for the development of biomarkers in this growing region of the world. Additionally, we were unable to include information on the longitudinal progression of CVD as well as diagnostic status and its influence on cognitive decline in the current study. This will be a key focus of our future studies. The availability of data over a 10-year period as well as the use of neuroimaging to define cerebrovascular disease is key strengths of our study.

## Conclusion

In conclusion, we demonstrate that there is a high frequency of YOD in our cohort with DAT and VaD being the most common in our clinic cohort. Younger patients with greater years of lifespan education and LOD patients with moderate-to-severe CVD experienced a steep decline in cognition.

## Supplementary Information

**Additional file 1: Supplementary Figure 1.** Number of young-onset and late-onset patients in a tertiary dementia clinic over 10 years. **Supplementary Figure 2.** Mini Mental State Examination score over time for young and late-onset dementia.

## Data Availability

The datasets used and/or analyzed during the current study are available from the corresponding author on reasonable request.

## Abbreviations

YOD: Young-onset dementia
LOD: Late-onset dementia
WMH: White matter hyperintensities
CVD: Cerebrovascular disease
VCI: Vascular cognitive impairment
DAT: Dementia of the Alzheimer’s type
FTD: Frontotemporal dementia
VaD: Vascular dementia
RPD: Rapidly progressing dementia
PDD: Parkinson’s dementia
DLB: Dementia with Lewy body
NPH: Normal pressure hydrocephalus
MMSE: Mini Mental State Examination
MoCA: Montreal Cognitive Assessment
GDS: Geriatric Depression Scale
