# Supplementary material for: Dementia in Southeast Asia: influence of onset-type, education, and cerebrovascular disease
Source: Alzheimers Res Ther. 2021 Nov 30;13:195. doi: 10.1186/s13195-021-00936-y (PMC8630908; doi:10.1186/s13195-021-00936-y)
Supplement: Supplementary file 1 — Additional file 1: Supplementary Figure 1. Number of young-onset and late-onset patients in a tertiary dementia clinic over 10 years. Supplementary Figure 2. Mini Mental State Examination score over time for young and late-onset dementia. [file 13195_2021_936_MOESM1_ESM.docx]

**SUPPLEMENTAL MATERIAL**


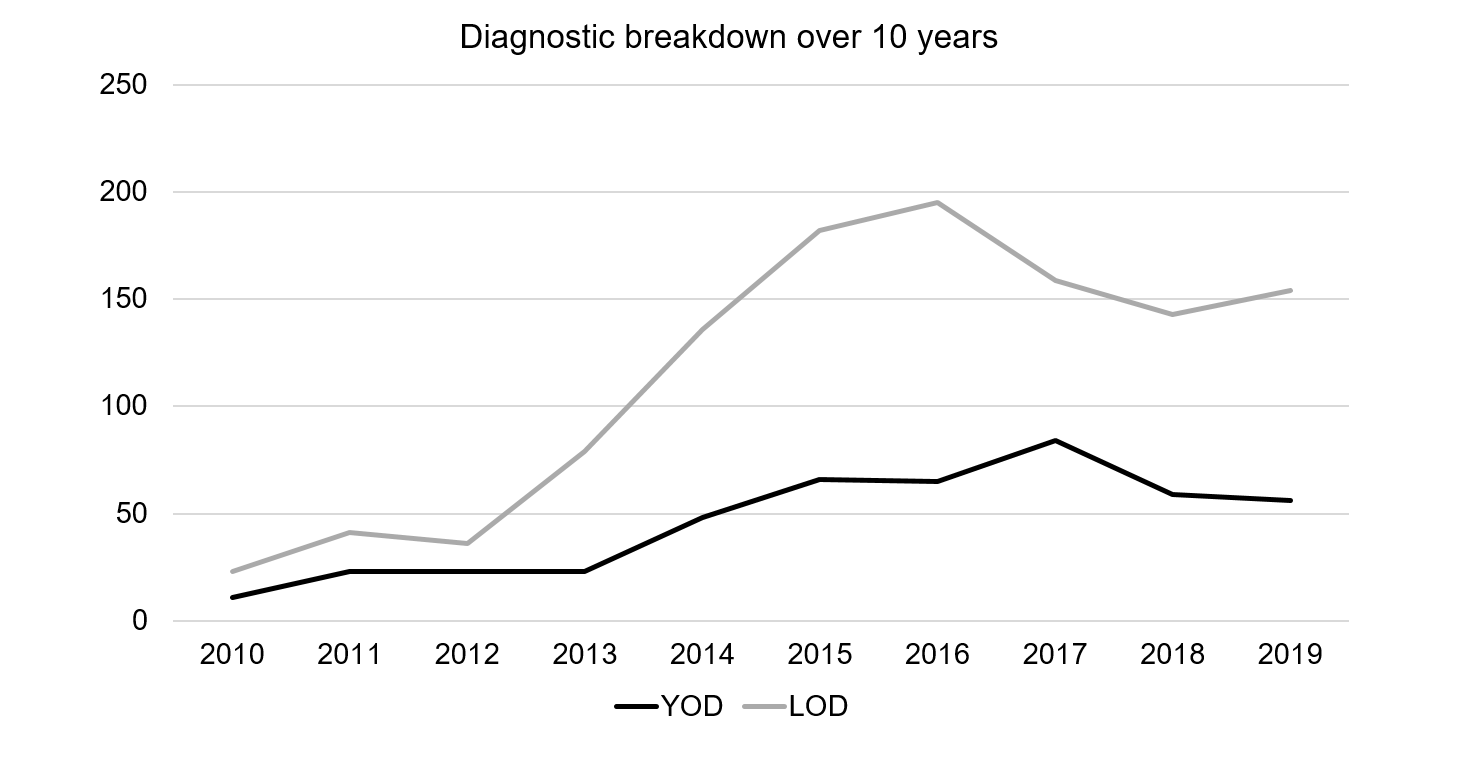


**Supplementary Figure 1. Number of young-onset and late-onset patients in a tertiary dementia clinic over 10 years.**

There was an increasing trend of patients in the young-onset and late-onset groups over the time period.

Abbreviations: YOD, young-onset dementia; LOD, late-onset dementia

**Supplementary Figure 2. Mini Mental State Examination score over time for young and late-onset dementia.**

Young-onset dementia patients had a higher MMSE score at baseline but showed a steeper decline in MMSE scores over time compared to the late-onset dementia group.

Abbreviations: YOD, young-onset dementia; LOD, late-onset dementia; MMSE, Mini Mental State Examination
